# Discriminating Between Attribute, Item-Position, and Wording Effects by the Congeneric and Tau-Equivalent Confirmatory Factor Analysis Models

**DOI:** 10.1177/00131644261419028

**Published:** 2026-03-11

**Authors:** Karl Schweizer, Xuezhu Ren, Tengfei Wang

**Affiliations:** 1 Goethe University Frankfurt, Germany; 2 Huazhong University of Science & Technology, Wuhan, China; 3 Zhejiang University, Hangzhou, China

**Keywords:** confirmatory factor analysis, congeneric model, tau-equivalent model, method effect

## Abstract

The capability of confirmatory factor analysis to discriminate common systematic variation of attribute, item-position, and wording effects was investigated using the congeneric and tau-equivalent models. The simulated data generated according to four approaches included gradually increased amounts of item-position or wording effect variation while the amount of attribute variation was kept constant. The congeneric model always signified good model fit independently of the type and amount of additional common systematic variation, that is, there was no discrimination. In applications of the tau-equivalent model, the increase of the item-position or wording effect variation led to the change from indicating good fit to bad model fit, that is, there was negative discrimination. In contrast, the additionally considered two-factor tau model discriminated positively. As a consequence of these results, we recommend the pre-screening of data for method effects.

## Introduction

This research explores how well the congeneric and tau-equivalent confirmatory factor analysis (CFA) models are suited for investigating the structure of data with and without a method effect. In data without a method effect, reaching a good model fit means that the latent variable of the CFA model representing the attribute of interest fully accounts for the common systematic variation of data. When a method effect is present, reaching a good model fit requires that the CFA model accounts for two types of common systematic variation, since in this case the variation of the attribute is accompanied by variation of the method effect, meaning overall inhomogeneity. The standard CFA model that is the congeneric CFA model (Brown, 2015; Graham, 2006) implicitly assumes that, given completed item selection, only one type of common systematic variation exists, and therefore, the latent variable representing the attribute of interest is sufficient to account for this variation.

The logic associating a specific type of common systematic variation with a corresponding latent variable suggests that a further latent variable is required when the data include additional common systematic variation of a method effect. This further latent variable can be the method-effect latent variable in a CFA model adapted to a multitrait-multimethod (MTMM) design (Byrne, 2016) or a target-specific latent variable that is a latent variable accounting for a specific type of common systematic variation (Zeller, Reiss, et al., 2017). MTMM-adapted CFA is not further considered since there are method effects evading purposeful manipulation necessary for reaching a complete MTMM design.

Method effects potentially undermining the suitability of the congeneric CFA model are the item-position effect (e.g., Ren et al., 2014; Sun et al., 2019; von Gugelberg et al., 2021) and the wording effect (e.g., DiStefano et al., 2012; DiStefano & Motl, 2006) since they are known to include common systematic variation of their own. The item-position effect increases from the first to the last items of the scale. This means that an increasing amount of common systematic variation from the first to last items is added to the common systematic variation of the attribute. The wording effect is observed when a scale includes positively and negatively worded items, creating additional common systematic variation. This effect is assumed to stem from processing reversed item wording (DiStefano et al., 2012; DiStefano & Motl, 2006) or alternatively from inconsistency in responding to negatively worded items (Arias et al., 2020, 2022, 2024).

Given the situation that these method effects are established empirical facts, the question arises how well the congeneric CFA model is prepared for dealing with different types of common systematic variation. Does it assure that the latent variable only accounts for common systematic variation of the attribute instead of common systematic variation in general? A basic characteristic of this CFA model calls the restriction to one type of common systematic variation into question: the free factor loadings (Graham, 2006). Free factor loadings mean that the estimation method exercises the major influence on what is represented by the latent variable. For example, when using the maximum likelihood method as an estimation method, it is the maximum likelihood criterion together with the expectation-maximization algorithm (Rubin & Thayer, 1982, p. 71f) that guides the estimation of factor loadings. We do not clearly see that this algorithm conducts maximization according to the attribute of interest instead of according to the set of available manifest variables in general.

Many simulation studies have explored the appropriateness of the congeneric CFA model for data with special properties, such as scale levels (e.g., DiStefano, 2002; Edwards et al., 2012; Li, 2016, 2021) and deviations from normality and range (e.g., Franco-Martinez et al., 2022; Jobst et al., 2021; Lai, 2018; West et al., 1995). Most of these studies have in common that the CFA model for investigating the data includes a single latent variable that is expected to account for the complete common systematic variation, as is represented by the covariance matrix model (Jöreskog, 1970). To date, the standard one-factor CFA model appears to be widely regarded as adequate for the investigation of the structural validity of scales in test construction and evaluation (e.g., Finney et al., 2016; Raykov, 2012).

Posing the same question (see the second to previous paragraph) with respect to the tau-equivalent CFA model leads to a different outcome. This CFA model, rooted in classical test theory (Graham, 2006; Lord & Novick, 1968, p. 47) includes the equivalence restriction for factor loadings that prevents the maximization of the amount of explained variation by modifying the pattern of factor loadings. While their general size may vary, they have to correspond to each other. Although recently it has become acceptable to substitute equal-sized factor loadings by patterns of factor loading replacements (Duncan & Duncan, 2004; Ferrer & McArdle, 2004; Zeller, Krampen, et al., 2017), estimation restricted to the pattern as a whole is still the basic characteristic of this CFA model. A downside of this model is that it displays sensitivity to deviations from tau equivalence that may result in model misfit in the absence of method effects.

The research reported in this paper addresses the question of whether one-factor congeneric and tau-equivalent CFA models actually account for the common systematic variation of the attribute and nothing else in the presence and absence of the item-position and wording effects. A two-factor model is additionally considered to check the presence of method-effect variation in the simulated data. The data generated for this purpose combine constant amounts of attribute variation with different amounts of method-effect variation.

### The One-Factor CFA Models

Given data collected by *p* items of a scale, the one-factor CFA model for structural investigations of centered data implicitly assumes a unidimensional data structure and is composed of two components: one representing the attribute of interest and the other representing influences that are unique for each manifest variable (Bollen, 1989, p. 18). Besides this basic CFA model, there is an extended version that also includes item-specific intercepts (Graham, 2006; Miller, 1995; Raykov, 1997). These intercepts are particularly important when analyzing mean differences between groups or between different measurement occasions. However, since the focus of this paper is only on investigating the structure of data, we omit the extended version.

Let $x\in {ℜ}^{p\times 1}$ be the *p *× 1 vector of centered manifest variables representing observations, $\lambda \in {ℜ}^{p\times 1}$ as **λ**_attribute_ be the *p* × 1 vector of factor loadings representing the relationships between the attribute latent variable and manifest variables, *ξ*_attribute_ be the latent variable representing the attribute, and $\delta \in {ℜ}^{p\times 1}$ be the *p* × 1 vector of residuals representing influences that are unique for each manifest variable. Then the basic version of the one-factor CFA model is given by

(1) $$x=\lambda_{\mathrm{attribute}}\xi_{\mathrm{attribute}}+\delta$$

(Brown, 2015). Vectors λ_attribute_ and **δ** include variables, which, as parameters, are free for estimation but can also be fixed according to theory-based expectations. Measurement models differ because of different specifications of λ_attribute_ and **δ**.

#### The Congeneric CFA Model

The major characteristic of this model is that it includes free factor loadings for reflecting the specific relationships between the individual manifest variables and the latent variable (Brown, 2015; Jöreskog, 1971). These characteristics allow the measurement model to reflect specificities of all manifest variables in a manner similar to the common-factor model (Lawley & Maxwell, 1971). To emphasize the variability of relationships of manifest and latent variables, we replace λ_attribute_ of Equation 1 with ω_attribute_ ( $\omega \in {ℜ}^{p\times 1}$ ) for the congeneric CFA model so that

(2) $$x=\omega_{\mathrm{attribute}}\xi_{\mathrm{attribute}}+\delta .$$

We selected the symbol omega for the free factor loadings to establish an association with the free factor loadings serving as input for the computation of the omega coefficient (McDonald, 1999). The latent variable of this model is referred to as *congeneric latent variable*.

#### The Tau Family of CFA Models

We classify CFA models as members of the tau family of CFA models if it is possible to conceive a factor loading on the latent variable as the product of the general parameter, τ∈ℜ , and a constant from the set of constants reflecting the basic assumption of the model.

##### The Tau-Equivalent CFA Model

This CFA model has emerged from the true-score theory of classical test theory (Alwin & Jackson, 1980; Lord & Novick, 1968, p. 47). It assumes equally sized elements of λ_attribute_, meaning equal relationships of manifest and latent variables. To highlight this specification, λ_attribute_ of Equation 1 is replaced by *τ *×**1** ( τ∈ℜ ):

(3) $$x=\tau_{\mathrm{attribute}}\times 1\times \xi_{\mathrm{attribute}}+\delta$$

where *τ*_attribute_ is the general factor loading, **1** is the *p* × 1 unity vector representing the equality assumption of tau equivalence, and δ is the vector of residuals. The restriction of parameter estimation to the general factor loading means the restriction of the degree to which the model can account for the common systematic variation of data. In contrast, the parameters in δ are estimated individually so that unique systematic variation cannot lead to a bad model fit. The latent variable of this model is referred to as *tau latent variable*.

##### The Tau-Proportional CFA Models

The restriction of factor loadings is also a characteristic of these models (e.g., Gignac, 2016; Schweizer, 2006; Tsai & Tsay, 2010), as is the case with the tau-equivalent model. Such factor loadings serve different purposes, one of which is representing experimental levels. The assumed pattern of relationships among such levels is mirrored by a corresponding pattern of relationships of manifest and latent variables. For example, using FLM (Schweizer, 2006), a subset of cognitive processes being stimulated once in the first level, twice in the second level and so on is represented by a vector with numbers one and two and so on as elements.

In tau-proportional CFA models, assumed relationships are represented by numbers assigned to the slots of the *p* × 1 vector of replacements of factor loadings, $\beta_{\mathrm{attribute}}\in {ℜ}^{p\times 1}$ , which replaces the unity vector of Equation 3. This modification gives

(4) $$x=\tau_{\mathrm{attribute}}\times \beta_{\mathrm{attribute}}\times \xi_{\mathrm{attribute}}+\delta .$$

This model is suitable for investigating method effects that require the consideration of characteristics extending across all items (or many of them) and include change. An example is the item-position effect that gradually increases along a series of items associated with the same attribute (Zeller, Krampen, et al., 2017). The latent variable of this model is referred to as *tau latent variable.*

### The Preparedness for Inhomogeneous Common Systematic Variation

The covariance matrix model used in CFA according to the model-fit approach (Gumedze & Dunne, 2011; Jöreskog, 1970) only distinguishes between common systematic variation and unique systematic variation but not between different types of common systematic variation. This model is defined as

(5) Σ=λϕλ'+θ

where $\Sigma \in {ℜ}^{p\times p}$ is the *p*×*p* model-implied covariance matrix, $\lambda \in {ℜ}^{p\times 1}$ is the *p*× 1 vector of factor loadings on the latent variable, $\phi \in {ℜ}^{\geq 0}$ is the variance parameter, and $\Theta \in {ℜ}^{p\times p}$ is the *p*×*p* diagonal matrix of residual variances. The first summand accounts for the common systematic variation of data, and the second one for the unique systematic variation.

Specifying the first summand of Equation 5 according to the congeneric model (Brown, 2015; Jöreskog, 1971) of Equation 2 leads to

(6) $$\lambda \phi \lambda^{\prime }=\omega_{\mathrm{attribute}}\phi \omega_{\mathrm{attribute}}\prime$$

that additionally requires setting either *ϕ* or *ω_i_* from {*ω*_1_, …, *ω_p_*} equal to one. A high degree of flexibility in accounting for common systematic variation characterizes this version of the model-implied covariance matrix. It is only slightly limited due to the necessity that each element of **ω** aligns with all other elements for reproducing the *p*×*p* empirical covariance matrix, **S**. This model is more likely to account for all (or almost all) common systematic variation of **S** instead of discriminating between different types of it.

The specification of the first summand of Equation 5 according to the tau-equivalent model (Equation 3) leads to

(7) $$\lambda \phi \lambda '=\left(\tau_{\mathrm{attribute}}\times 1\right)\phi \left(\tau_{\mathrm{attribute}}\times 1\right)\prime .$$

To simplify parameter estimation, the right-hand part of Equation 7 is reorganized so that

λϕλ'=1ϕ*1′

where ϕ* = *τ*_attribute_*ϕτ*_attribute_ so that only ϕ* needs to be estimated. In the case that there is scaling according to the reference group method (*ϕ* = 1), ϕ* = *τ*_attribute_^2^. This version of λϕλ' fits well with empirical covariance matrices that are basically uniform matrices (with the exception of the main diagonal). It is very unlikely that this model accommodates to inhomogeneous common systematic variation.

The tau-proportional models (Equation 4) with pre-specified relationships between the manifest and latent variables but without the equality restriction can account for many patterns of common systematic variation. The specification of the first summand of the covariance matrix model (Equation 5) according to these measurement models is given by

(8) $$\lambda \phi \lambda '=\left(\tau_{\mathrm{attribute}}\times \beta \right)\phi \left(\tau_{\mathrm{attribute}}\times \beta \right)'.$$

Again, to simplify parameter estimation, Equation 8 is reorganized such that

$$\lambda \phi \lambda '=\beta \phi^{*}\beta '$$

where ϕ* = *τ*_attribute_*ϕτ*_attribute_ so that only ϕ* needs to be estimated (see also Equation 7). Because of the large number of possible patterns of pre-specified (proportional) relationships, these models can account for a larger number of covariance matrices than the tau-equivalent model. However, the confirmatory approach does not allow for adaptation to unexpected inhomogeneity of common systematic variation.

### The Approaches for Data Simulation

Four approaches for the generation of structured random data are described, each of which plays a role in the empirical section. Two of them yield data including one type of common systematic variation only (the traditional and realistic approaches), while the other two yield data including two types of common systematic variation (the position-reflecting and wording-effect approaches).

The *traditional approach* in simulation research proceeds from the assumption that the relationships between manifest and latent variables are constant across the columns of a data matrix. Data constructed accordingly are expected to exhibit factor loadings of equal sizes (*λ_i_*, *λ_j_*, *i*, *j*=1, …, *p, i≠j*):

$$\lambda_{i}=\lambda_{j}.$$

The *realistic approach* overcomes a possible disadvantage of the traditional approach, which assumes equal-sized relationships between manifest and latent variables, by allowing for internal inhomogeneity. This is realized by varying the relationships between neighboring columns of a data matrix so that factor loadings of different sizes emerge (Li, 2021). The specific pre-fixed pattern of relationships between neighboring columns of the data matrix (e.g., columns *i*, *j*, *k*, *l, i≠j, i≠k, j≠k*) is expected to lead to the following pattern of relationships of factor loadings:

$$\lambda_{i}<\lambda_{j},\lambda_{j}>\lambda_{k},\lambda_{k}<\lambda_{l}=\lambda_{i}.$$

The *position-reflecting approach* combines constancy in variation with a representation of the item*-*position effect. This effect has been frequently reported in investigating real data and has been demonstrated in simulated data using different analysis methods (e.g., Debeer & Janssen, 2013; Hartig & Buchholz, 2012; Lozano & Revuelta, 2020; Verguts & De Boeck, 2000; Zeller, Krampen, et al., 2017). The simulated data have to include an attribute contribution consisting in constant common systematic variation, giving when considered in isolation rise to equal-sized factor loadings (*λ*_(attribute)*i*_, *λ*_(attribute)*j*_, *i*, *j*=1, …, *p, i≠j*), and a contribution of the item-position effect consisting in gradually increasing common systematic variation, giving when investigated in isolation rise to linearly increasing factor loadings (*λ*_(method)*l*_, *λ*_(method)*k*_, *l*, *k*=1, …, *p, l≠k*):

$$\lambda_{\left(\mathrm{attribute}\right)i}=\lambda_{\left(\mathrm{attribute}\right)j}\mathrm{and}\lambda_{\left(\mathrm{method}\right)l}<\lambda_{\left(\mathrm{method}\right)k}.$$

The *wording-effect approach* conceptualized as effect due to reverse item wording combines overall constancy in variation with other constancy in variation restricted to a subset of manifest variables: one type of variation extending to all items, as is typical of attribute variation (*λ*_(attribute)*i*_, *λ*_(attribute)*j*_, *i*, *j*=1, …, *p, i≠j*), and other constant variation restricted to the negatively worded items, as is typical of the wording effect (*λ*_(method)*l*_*λ*_(method)*k*_, *l*, *k*= *p*/2 + 1, …, *p, l≠k*):

$$\lambda_{\left(\mathrm{attribute}\right)i}=\lambda_{\left(\mathrm{attribute}\right)j}\;\mathrm{and}\;\lambda_{\left(\mathrm{method}\right)l}=\lambda_{\left(\mathrm{method}\right)k}$$

(for negative worded items only) (DiStefano et al., 2012; DiStefano & Motl, 2006).

For more details, see the “Method” and the “Results” sections.

### Objectives of the Empirical Investigation

The empirical investigation explored how well the one-factor congeneric and tau-equivalent CFA models performed under conditions requiring accounting for inhomogeneous data variation. The two-factor tau CFA model was additionally considered to check whether the data included the expected two different types of common systematic variation. The reasons for inhomogeneity were internal variability and the combination of two different types of variation. The data generation followed the four simulation approaches and included five inhomogeneity levels each.

Following conventions of test construction and evaluation, the expectation was that the latent variable of the one-factor CFA model would account for the common systematic variation of the attribute and nothing else. But, in the presence of additional common systematic variation, this latent variable could also accounted for this additional variation, meaning good model fit, or fail to do so, meaning model misfit. In the first case, the additional common systematic variation would be treated as variation of the attribute, meaning no discrimination of types of variation. In the second case, the latent variable would correctly account for the common systematic variation of the attribute but yield misfit because of the additional unaccounted common systematic variation. We refer to this situation as negative discrimination. If the two latent variables of the two-factor CFA model correctly accounted for the different types of common systematic variation that could be assured by differing constraints of the factor loading replacements of the two latent variables, we refer to it as positive discrimination.

## Method

The general characteristics of generating and investigating simulated data are outlined in this section, while the specificities of the four data conditions according to the four approaches and the corresponding results are reported in the following four sections. Data generation was accomplished using two methods: (1) following the method described by Jöreskog and Sörbom (2001), making use of relational patterns, or (2) creating random data [*N*(0,1)] in the first step and using a linear equation for combining them in the second step. These generation methods assured that there were independent types of common systematic variation. Each data type included five treatment levels, with each level comprising 500 matrices, and each generated data matrix comprised 500 rows and 13 columns.

The CFA models for investigating the data structure followed Equations 2 (congeneric model) and 3 (tau-equivalent model). The congeneric CFA model required the estimation of the factor loadings in combination with the variance parameter fixed to one (reference-group scaling method for the factor variance). In contrast, the tau-equivalent and tau CFA models included factor loading replacements (see below) while the variance parameters were estimated (for an explanation, see Equations 7 and 8). The scaling of the factor variance occurred according to the criterion-based method. The data are stored in the OSF repository.

The two-factor tau CFA models included two latent variables to target the two expected types of common systematic variation. They were realized as

(9) $$x=\tau_{\mathrm{attribute}}\times 1\times \xi_{\mathrm{attribute}}+\tau_{\mathrm{method}\_\mathrm{effect}}\times \beta_{\mathrm{method}\_\mathrm{effect}}\times \xi_{\mathrm{method}\_\mathrm{effect}}+\delta$$

where $x\in {ℜ}^{p\times 1}$ was the *p* × 1 vector of centered manifest variables,**** $\tau_{\mathrm{attribute}}\in ℜ$ and $\tau_{\mathrm{method}\_\mathrm{effect}}\in ℜ$ were general factor loadings, **1** and **β**_method_effect_ were pre-specified *p* × 1 vectors including factor loading replacements, *ξ*_attribute_ and *ξ*_method_effect_ were latent variables, and $\delta \in {ℜ}^{p\times 1}$ was the *p* × 1 vector of residuals. **1** represented tau equivalence and **β** the pattern characterizing the targeted method effect. The first summand corresponded to the first summand of Equation 3 and the second summand to the first summand of Equation 4.

When mimicking the item-position effect, the factor loading replacements on the position-effect latent variable started from zero and linearly increased while in mimicking the wording effect, they were constant. The exact sizes of factor loading replacements were available after preparation for scaling. Note. Scaling does not influence model fit in ML estimation.

The estimation method was maximum likelihood estimation using the LISREL software package (Jöreskog & Sörbom, 2006) with covariance matrices computed from simulated data as input. The following fit indices were recorded and evaluated (established fit cutoffs are included in parentheses): RMSEA (≤0.06), SRMR (≤0.08), NNFI (≥0.95), and CFI (≥0.95; see DiStefano, 2016; Hu & Bentler, 1999). Following established traditions, *χ*^2^, *df*, and AIC were also provided, while the focus in evaluation was on fit indices (Goretzko et al., 2023). Means (and standard deviations) were computed and included in tables and figures. Results for models and levels were compared using the CFI difference (0.01) and the RMSEA difference (0.015; Cheung & Rensvold, 2002). To check whether both latent variables of the two-factor CFA model accounted for a substantial amount of common systematic variation, the factor variances were scaled (Schweizer et al., 2019, Equation 15) and evaluated.

## Results

### The Results of the Traditional Data Condition

The relational patterns for generating the data consisted in equal-sized diagonal elements and also equal-sized off-diagonal elements. The diagonal elements were ones, while the off-diagonal elements reflected the treatment levels. The sizes according to these levels were chosen to be reproducible by factor loadings of 0.275 (level 1), 0.350 (level 2), 0.425 (level 3), 0.500 (level 4), and 0.625 (level 5). The models for data analysis were the one-factor congeneric and tau-equivalent CFA models.

The mean fit results and standard deviations are reported in Table 1. The first part includes the results for the congeneric model and the second one the results for the tau-equivalent model. The smaller third part provides the differences between the level means reported in the first and second parts. The means of all levels (first and second parts) indicated good model fit according to the cutoffs. SRMR, NNFI and CFI displayed a minor numerical improvement in fit within the models across the levels. There were minor differences between the fit results for the two models (third part), but neither an RMSEA difference nor a CFI difference reached the cutoffs for differences. The smaller *χ*^2^s characterized the congeneric model and the smaller AICs the tau-equivalent model. In sum, there were no substantial differences between the two CFA models.

**Table 1.** Means and Standard Deviations of Fit Estimates Obtained by One-Factor Congeneric and Tau-Equivalent CFA Models in Data According to the Traditional Approach (*N* = 500).

Model type	Level	Statistic	χ^2^	df	RMSEA	SRMR	NNFI	CFI	AIC
Congeneric	1	Mean	65.42	65	0.008	0.035	0.998	0.976	117.42
(Co)		*SD*	11.27		0.009	0.003	0.070	0.036	11.57
	2	Mean	65.51	65	0.008	0.033	0.998	0.991	117.51
		*SD*	11.65		0.009	0.003	0.025	0.013	11.65
	3	Mean	65.50	65	0.008	0.031	0.999	0.996	117.50
		*SD*	11.70		0.009	0.003	0.011	0.006	11.7
	4	Mean	65.46	65	0.008	0.028	0.999	0.998	117.46
		*SD*	11.75		0.009	0.003	0.006	0.003	11.75
	5	Mean	65.39	65	0.008	0.023	1.000	0.999	117.39
		*SD*	11.80		0.009	0.002	0.002	0.001	11.80
Tau	1	Mean	77.12	77	0.007	0.041	0.999	0.976	105.11
(Ta)		*SD*	12.23		0.009	0.003	0.061	0.036	12.23
	2	Mean	77.10	77	0.007	0.041	0.999	0.991	105.10
		*SD*	12.26		0.009	0.004	0.022	0.013	12.26
	3	Mean	77.10	77	0.007	0.041	0.999	0.996	105.10
		*SD*	12.28		0.009	0.004	0.010	0.007	12.28
	4	Mean	77.11	77	0.007	0.040	1.000	0.998	105.11
		*SD*	12.29		0.009	0.004	0.005	0.003	12.29
	5	Mean	77.13	77	0.007	0.039	1.000	0.999	105.13
		*SD*	12.29		0.009	0.005	0.002	0.001	12.30
Ta—Co	1		11.70	12	−0.001	0.006	0.002	−0.000	−12.30
	2		11.59	12	−0.001	0.008	0.001	−0.000	−12.40
	3		11.60	12	−0.001	0.010	0.000	−0.000	−12.40
	4		11.64	12	−0.001	0.012	0.000	−0.000	−12.36
	5		11.75	12	−0.001	0.016	0.000	−0.000	−12.25

### The Results of the Realistic Data Condition

**Figure 1.**
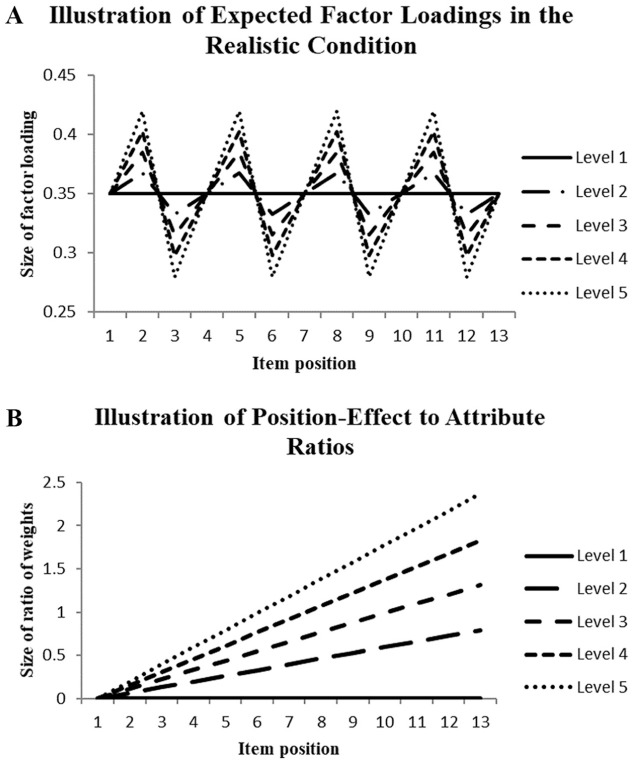
Illustration of the Variation of Relationships of Items and Latent Dimension Characterizing the Realistic Approach (A) and the Ratio of Method-Effect and Attribute Weights Characterizing the Item-Position Effect (B) Across the Item Positions.

This investigation used data with varying relationships between neighboring columns of the data matrix, which fits better with the congeneric CFA model than the tau-equivalent CFA model. The data simulation assured that the expected factor loadings for the first, fourth, seventh and so on columns were 0.35. The expected size of the factor loading for the first one of the in-between columns was increased and for the second one decreased using linear equations with independent random variables [*N*(0,1)]. Different percentages of increases and decreases of the in-between columns were realized (0, 5, 10, 15, and 20%), resulting in five treatment levels. Figure 1A illustrates the deviations from the selected mean factor loadings across five treatment levels.

This way of data generation ensured overall constancy in combination with variability in the near neighborhood of each column. The same congeneric and tau-equivalent CFA models as used in investigating the first data type were employed in the structural investigation.

Table 2 reports the mean fit results and standard deviations. As in Table 1, the first part includes the results for the congeneric CFA model and the second part includes the results for the tau-equivalent CFA model. The smaller third part includes the differences between the level means. The congeneric model (first part) yielded estimates of constant size, indicating good model fit across all levels. In contrast, for the tau-equivalent model (second part), the results of the fit indices with a cutoff indicated an overall decrease in model fit from the first to fifth levels, although good model fit was indicated in all levels. In the third part, an increasing degree of difference between the models was indicated. In the last level (level 5), the CFI difference (0.017) reached significance, while the RMSEA difference (0.014) was almost significant. Overall, the best performance was observed for the congeneric CFA model.

**Table 2.** Means and Standard Deviations of Fit Estimates Obtained by One-Factor Congeneric and Tau-Equivalent CFA Models in Data According to the Realistic Approach (*N* = 500).

Model type	Level	Statistic	χ^2^	df	RMSEA	SRMR	NNFI	CFI	AIC
Congeneric	1	Mean	65.08	65	0.007	0.032	0.999	0.993	117.08
(Co)		*SD*	11.95		0.009	0.003	0.021	0.012	11.95
	2	Mean	65.11	65	0.007	0.032	0.999	0.995	117.11
		*SD*	11.93		0.009	0.003	0.015	0.008	11.93
	3	Mean	65.11	65	0.007	0.032	0.999	0.995	117.11
		*SD*	11.92		0.009	0.003	0.015	0.008	11.92
	4	Mean	65.11	65	0.007	0.032	0.999	0.995	117.11
		*SD*	11.91		0.009	0.003	0.015	0.008	11.91
	5	Mean	65.11	65	0.007	0.031	0.999	0.995	117.11
		*SD*	11.90		0.009	0.003	0.015	0.008	11.90
Tau	1	Mean	75.26	77	0.007	0.041	0.997	0.992	105.26
(Ta)		*SD*	17.57		0.009	0.005	0.046	0.045	14.36
	2	Mean	78.52	77	0.008	0.042	0.998	0.993	106.52
		*SD*	13.62		0.009	0.005	0.014	0.010	13.62
	3	Mean	82.45	77	0.011	0.044	0.994	0.991	110.45
		*SD*	14.42		0.010	0.005	0.015	0.012	14.42
	4	Mean	89.09	77	0.015	0.048	0.987	0.986	117.09
		*SD*	15.67		0.011	0.006	0.016	0.014	15.67
	5	Mean	98.56	77	0.022	0.053	0.978	0.978	126.56
		*SD*	17.37		0.010	0.006	0.017	0.016	17.37
Ta—Co	1		10.17	12	−0.000	0.009	−0.002	−0.001	−11.85
	2		13.42	12	0.001	0.010	−0.001	−0.001	−10.58
	3		17.35	12	0.003	0.013	−0.005	−0.004	−6.65
	4		23.98	12	0.008	0.017	−0.012	−0.009	−0.02
	5		33.44	12	0.014	0.022	−0.021	−0.017	9.44

### The Results of the Position-Reflecting Data Condition

Structured random data were generated that consisted of a component according to the traditional approach reflecting the attribute influence and a component mimicking the item-position effect. In the construction of data, the weight for the position-effect component of the linear equation was increased across levels while the weight for the other component was kept constant, resulting in the following ratios of weights (numerator: item-position weight; denominator: attribute weight) in the last column of a data matrix: 0 in level 1, 0.6 in level 2, 1.2 in level 3, 1.8 in level 4 and 2.4 in level 5. Figure 1B provides an illustration of the weight ratios employed in data generation across the 13 columns of a data matrix. The data were investigated using one-factor congeneric and tau-equivalent CFA models as well as the two-factor tau CFA model. While the one-factor CFA models should signify model misfit for large weight ratios, the two-factor CFA model should indicate good model fit.

**Figure 2.**
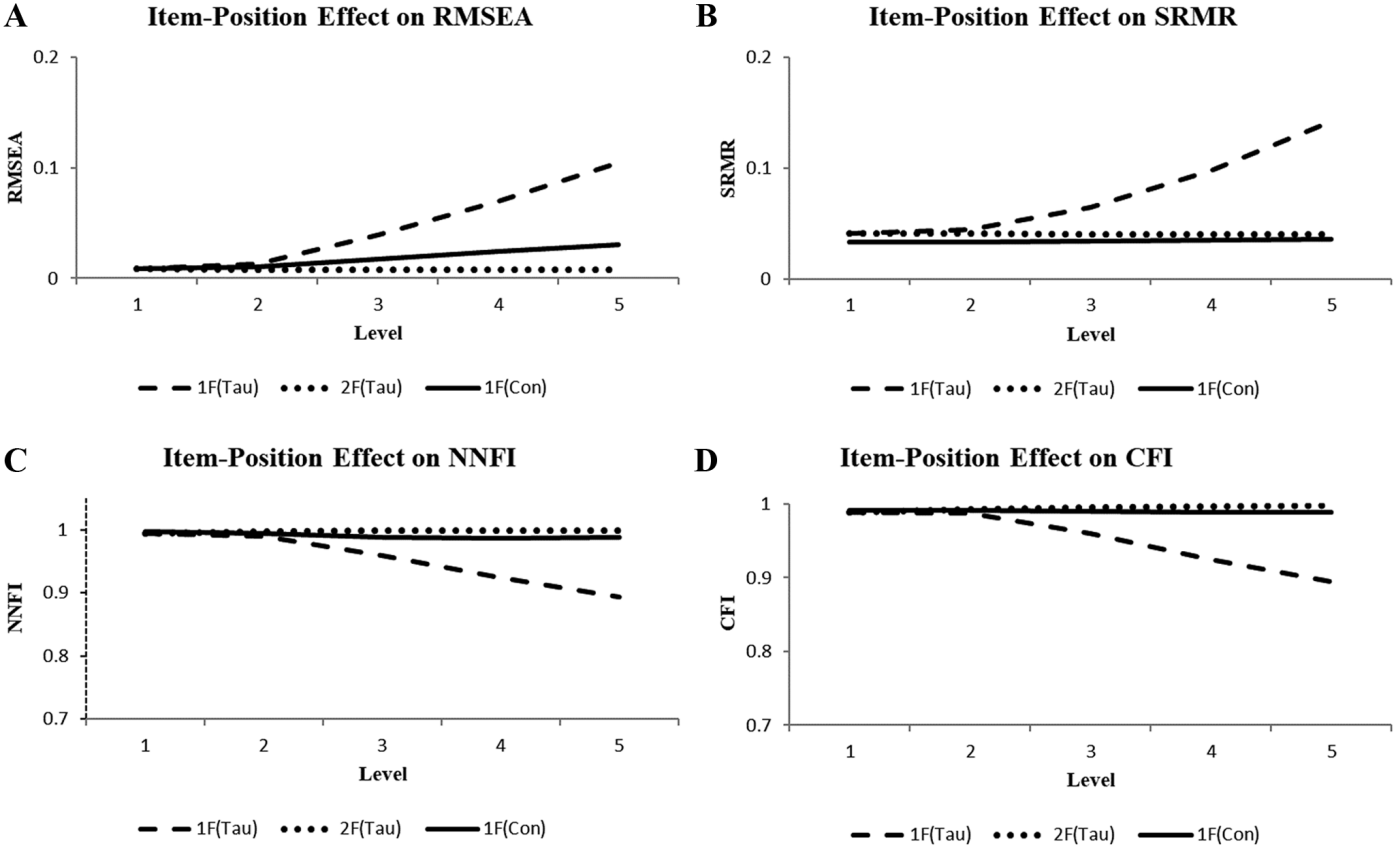
Illustrations of the Item-Position Effect on RMSEA (A), SRMR (B), NNFI (C), and CFI (D) Observed Using the One-Factor Tau-Equivalent [1F(Tau)] and Congeneric [1F(Con)] Models as well as the Two-Factor Tau-Based [2F(Tau)] Models Across Five Levels.

The fit results for the fit indices with a cutoff are illustrated in Figure 2. Depicted as solid curves, the illustrations of the results of the one-factor congeneric CFA model [1F(Con)] were virtually straight lines, indicating no fit impairment in all fit indices with a cutoff (2A, 2B, 2C, 2D), despite the increasing amount of the item-position effect. In contrast, all dashed lines representing the results of the one-factor tau-equivalent CFA model changed from initially indicating good model fit in the direction of indicating bad model fit [1F(Tau)]. The RMSEA, SRMR, NNFI, and CFI estimates of levels 4 and 5 were beyond the corresponding cutoffs. Further, as expected, the two-factor tau CFA model [2F(Tau)] yielded indications of good model fit according to all fit indices with a cutoff across all levels (see dotted lines). The scaled estimates of factor variances of level 5 were 3.64 (*SD* = 0.47) and 4.32 (*SD* = 0.57) for attribute and position-effect latent variables in corresponding order. Each mean differed from zero by more than two standard deviations.

In sum, the shift from indicating good model fit to bad model fit observed for the one-factor tau-equivalent CFA model implicitly signified the increasing presence of additional common systematic variation. The results for the two-factor tau CFA model also indicated the presence of two types of common systematic variation. In contrast, the one-factor congeneric CFA model constantly signified the presence of only one type of common systematic variation.

### The Results of the Wording-Effect Data Condition

The data matrices generated to include the wording effect comprised six columns (1–6) with common systematic variation of the attribute only and seven columns (7–13) with common systematic variation of both the wording effect and the attribute. In data generation by linear equations, the common systematic variation of the attribute was kept constant across all columns, while additional common systematic variation of the wording effect was also constant, but across the second half of columns only. Five treatment levels were generated by modifying the size of the wording effect, giving rise to the following ratios of weights (numerator: wording-effect weight; denominator: attribute weight): 0 in level 1, 0.15 in level 2, 0.30 in level 3, 0.45 in level 4, and 0.60 in level 5. The data were investigated using the one-factor tau-equivalent and congeneric CFA models as well as the two-factor tau CFA model. While the one-factor models should signify model misfit in investigating higher-level data, because of the wording, the two-factor model was expected to always indicate good model fit.

**Figure 3.**
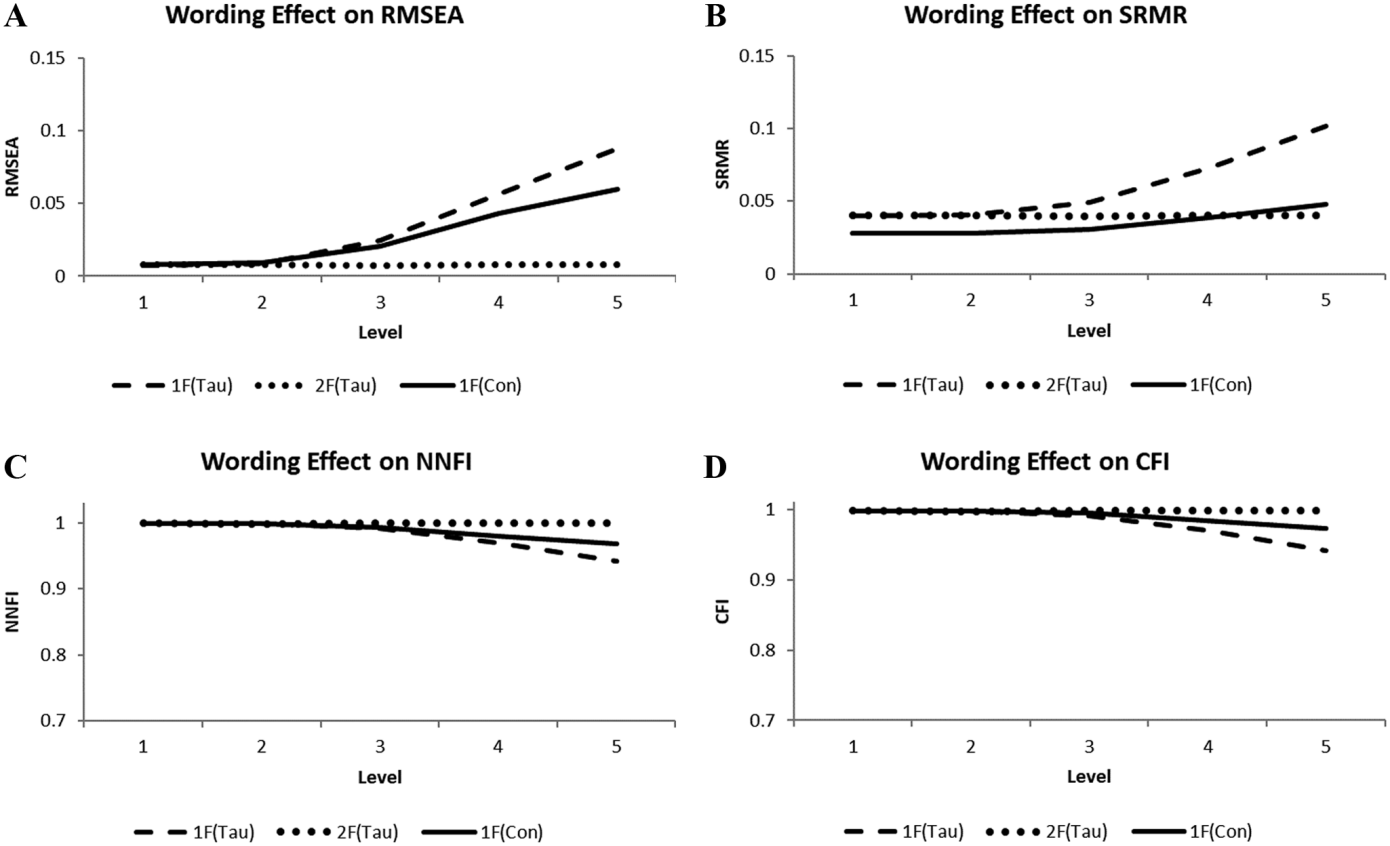
Illustrations of the Wording Effect on RMSEA (A), SRMR (B), NNFI (C), and CFI (D) Observed Using the One-Factor Tau-Equivalent [1F(Tau)] and Congeneric [1F(Con)] Models as well as the Two-Factor Tau-Based [2F(Tau)] Models Across Five Levels.

The curves of Figure 3 illustrate the fit results for the indices with a cutoff. The dotted curves representing the results of the two-factor tau CFA model [2F(Tau)] signified good model fit under all conditions (Figure 3A–D). In contrast, the dashed curves for the one-factor tau-equivalent CFA model [1F(Tau)] gradually bent away from the horizontal axis and ended up indicating a bad model fit for level 5 data (Figure 3A–D).

Regarding the one-factor congeneric CFA model [1F(Con)], the results were not as clear-cut as in the other conditions. As is apparent from the illustrations, the curves of the one-factor congeneric model [1F(free)] bent away from the horizontal axis when the amount of common systematic variation increased, indicating some degree of sensitivity for the wording effect. But the estimates always stayed in the range, indicating good model fit. Only the level-5 RMSEA result came close to the cutoff. The scaled estimates of factor variances for the two-factor tau CFA model in fifth-level data were 4.64 (*SD* = 0.47) and 2.51 (*SD* = 0.29) for attribute and wording-effect latent variables in corresponding order. Each mean was larger than two standard deviations.

In sum, the fit estimates for the one-factor tau-equivalent CFA model reflected the increasing amount of common systematic variation and ended up indicating model misfit in the fifth level, meaning negative discrimination, whereas the results for the one-factor congeneric CFA model always indicated good model fit, meaning no discrimination. In contrast, the two-factor tau model yielded a good fit and discriminated the types of common systematic variation as expected (see also Appendix).

## Discussion

While CFA research of the early days can be conceived as a search for the model with the latent variable accounting for the complete common systematic variation of the data (Jöreskog, 1970), nowadays, one focus of research is to assure that different types of common systematic variation are correctly discriminated by corresponding latent variables. One way of discriminating between different types of common systematic variation is associating latent variables with subsets of manifest variables, as is characteristic of CFA on the basis of MTMM designs (Byrne, 2016). In such CFA models, the factor loadings are freely estimated, that is, these models own congeneric characteristics. The other way of discriminating between different types of common systematic variation is to use target-specific latent variables accounting for targeted types of common systematic variation (Zeller, Reiss, & Schweizer, 2017). In such CFA models, patterns of factor loading replacements assure that the latent variables account for the corresponding types of common systematic variation. In these models, the fixture of the relationships among factor loadings replacements is inherited from the tau-equivalent CFA model.

The reasoning of the previous paragraph suggests that the congeneric CFA model may not be suited for the investigation of the structure of data in cases of insecurity regarding the type(s) of common systematic variation of data. The results of the simulation study regarding this model that originates from the early days of CFA research (Jöreskog, 1971) are twofold but generally in line with the suggestion. In data with one type of common systematic variation only, the model performs well and excels the tau-equivalent model when internal disturbance is also present, as in the case of the investigation of realistic data. But the congeneric CFA model also upholds the indication of good model fit despite the increasing presence of a second type of common systematic variation. This means that the congeneric model does not discriminate types of common systematic variation in the absence of a design enforcing discrimination.

It needs to be added that the construction of this CFA model was guided by the aim that the latent variable should account for the complete common systematic variation of the data characterizing a specific set of manifest variables, and performed well when compared with other models (Graham, 2006). Therefore, the application for discriminating between different types of common systematic variation outside of the MTMM framework is a new challenge to this CFA model that may require a pre-screening of data for method effects before its application. We recommend such pre-screening.

The tau-equivalent CFA model contrasts with the congeneric CFA model. The increasing presence of a second type of common systematic variation impairs model fit, which can be interpreted as discrimination between the two types of common systematic variation in a negative way. For discrimination in a positive way, a second latent variable must be included in the CFA model, as is also demonstrated in the simulation study. The two-factor CFA model enables the confirmation of the assumed structure (Vogt & Johnson, 2016) including discrimination between two types of common systematic variation.

What additionally needs to be mentioned is that impairment of model fit can occur because of internal disturbance, as in the case of the investigation of realistic data. The tau-equivalent CFA model is not only sensitive to method effects but also to a lack of consistency in the representation of the attribute. Inconsistency due to item particularities is likely to impair model fit. A less strict version of the tau-equivalent CFA model is necessary to overcome this disadvantage.

The four data conditions of the simulation study challenge the CFA models in different ways. But it is common to all data conditions that the first level includes no disturbing influence and that such influence is gradually increased. In the item-position and wording effect conditions, the last level is selected such that in at least one case the change to the indication of bad model fit is observed according to one of the fit indices with a cutoff (DiStefano, 2016; Hu & Bentler, 1999).

In the reported research, the wording effect is conceptualized as a common systematic variation due to the processing of negatively worded information. We acknowledge that it is not the sole conceptualization of this effect. There is an alternative conceptualization of this effect as inconsistency of responses due to careless/insufficient effort (Arias et al., 2020, 2022, 2024). A small fraction of inconsistent respondents may be the main reason for an observed wording factor. The reported results should not be generalized over both conceptualizations although it may be added that heterogeneity due to careless/ insufficient effort also suggests the presence of a kind of additional variation that may lead to model misfit in the application of the tau-equivalent CFA model.

The challenges of the two conditions including two types of common systematic variation consist in the demand to discriminate attribute-related common systematic variation from other types of common systematic variation. Considering these conditions as situations requiring the demonstration of structural validity by means of a one-factor CFA model is incorrect because one type of common systematic variation is purposefully neglected. Instead, it is necessary to demonstrate discriminant validity, contributing to construct validity (Messick, 1981) of the investigated scale. Using the congeneric model for this purpose requires an MTMM design as a framework that, however, as a symmetric structure, does not fit well with the generated situations. In contrast, as already indicated, the tau-equivalent one-factor CFA model can be employed for the discrimination of two types of common systematic variation but in a negative way only. Better discrimination in the sense of discriminant validity is achievable by the two-factor tau CFA model since, at the same time, the two types of common systematic variation are identified.

After scrutinizing the performance of the congeneric model and before closing this paper, it needs to be emphasized that the congeneric model is an important model and marks an important step in the investigation of the structure of data. It is useful for identifying sets of items potentially representing attributes, and in the absence of method effects, it provides the most robust estimate of the structural validity of a scale. What is needed for dealing with situations including two types of common systematic variation is the association with a check for the presence of method effects.

A limitation of the study is that only the two types of method effects that we consider as among the most important ones are entertained. More types of method effects might have provided a more complete picture of how the congeneric and tau-based CFA models perform. Another limitation is that complications such as variation in scale levels of data, sample size, and distributional specificities of data are not investigated.

In sum, the congeneric model discriminates between common systematic variation and unique systematic variation but not another type of common systematic variation unless in combination with an MTMM design. In contrast, tau-based models can distinguish between different types of common systematic variation in the absence of internal inconsistency when latent variables with appropriately specified factor loading replacements are available as one-factor models in a negative way and as two-factor models in a positive way.

## Appendix

**Table A1.** Percentages of Indications of Good Model Fit Observed in Investigating Data Including the Item-Position Effect and the Wording Effect.

Effect type	Model type	No. factors	Level	RMSEA	SRMR	NNFI	CFI
Position	Congeneric	1	1	100	100	97	98
effect		1	2	100	100	98	99
		1	3	100	100	99	99
		1	4	100	100	99	99
		1	5	99	100	99	100
	Tau	1	1	100	100	98	98
		1	2	100	100	98	98
		1	3	99	99	73	74
		1	4	14	2	5	5
		1	5	0	0	0	0
	Tau	2	1	100	100	98	98
		2	2	100	100	99	99
		2	3	100	100	99	99
		2	4	100	100	99	99
		2	5	100	100	100	100
Wording	Congeneric	1	1	100	100	100	100
effect		1	2	100	100	100	100
		1	3	100	100	100	100
		1	4	98	100	100	100
		1	5	47	100	96	99
	Tau	1	1	100	100	100	100
		1	2	100	100	100	100
		1	3	100	100	100	100
		1	4	70	83	98	98
		1	5	15	17	28	30
	Tau	2	1	100	100	100	100
		2	2	100	100	100	100
		2	3	100	100	100	100
		2	4	100	100	100	100
		2	5	100	100	100	100
